# Investigating disease awareness of cutaneous leishmaniasis in rural Sri Lanka to inform public health services: a cross-sectional study

**DOI:** 10.1136/bmjopen-2024-088714

**Published:** 2024-11-24

**Authors:** Sonali Dinushika Gunasekara, Thilini Chanchala Agampodi, Manjula Weerasinghe, Manoj Sanjeewa Fernando, Helen Philippa Price, Nuwan Darshana Wickramasinghe, Suneth Buddhika Agampodi

**Affiliations:** 1 Department of Community Medicine, Faculty of Medicine and Allied Sciences, Rajarata University of Sri Lanka, Saliyapura, Sri Lanka; 2 Department of Health Promotion, Faculty of Applied Sciences, Rajarata University of Sri Lanka, Mihintale, Sri Lanka; 3 School of Life Sciences, Keele University, Newcastle-under-Lyme, Staffordshire, UK; 4 International Vaccine Institute, Gwanak-gu, Seoul, Republic of Korea; 5 Section of Infectious Diseases, Department of Internal Medicine, School of Medicine, Yale University, New Haven, Connecticut, USA

**Keywords:** Neglected Diseases, Awareness, Cross-Sectional Studies, Surveys and Questionnaires, INFECTIOUS DISEASES

## Abstract

**Abstract:**

**Objective:**

To assess community awareness of cutaneous leishmaniasis (CL) in a disease-endemic district in Sri Lanka.

**Design:**

Population-based cross-sectional study.

**Setting:**

This study was conducted in selected 158 Grama Niladhari divisions covering all the 22 Divisional Secretariat areas of the Anuradhapura district, Sri Lanka.

**Sampling technique and participants:**

A probability sample of households was selected using multistage cluster sampling. Adults (≥18 years) who resided permanently in the Anuradhapura district during the data collection period were eligible, and individuals who could not comprehend or respond to the questions were excluded. The primary or secondary healthcare-related decision-maker of 1555 households participated in the study, in which 1479 (95.1%) were Sinhalese in ethnicity, including 1157 (74.4%) females.

**Primary and secondary outcome measures:**

The primary outcome measure was ‘CL awareness,’ operationalised by awareness of the disease name, transmission mode and the primary disease characteristic based on a systematic expert consensus approach. Secondary outcome measures included awareness and perceptions of CL curability, treatment centres and perceived susceptibility.

**Results:**

Only 3.6% (n=56) demonstrated CL awareness based on the definition. We observed low disease awareness even among people who claimed having CL or previously had the disease (n=6, 27.3%). While 1065 (68.5%) had heard the correct name (‘leishmaniasis’) or the local name (*‘wæli mæssāgē leḍē’*) for the disease, only 224 (21.0%) out of that knew the mode of transmission and 128 (12.0%) the chronic nature of CL skin lesions, respectively. Among the respondents with CL awareness (n=56), 42 (75.0%), 54 (96.4%) and 44 (88.0%) perceived CL as curable, were aware of treatment centres and expressed self-perceived susceptibility, respectively. Despite 423 (46.4%) who claimed to know more beyond the disease name, only 35 (8.3%) demonstrated actual CL awareness.

**Conclusion:**

Findings highlight significantly low CL awareness in the disease-endemic Anuradhapura district, and we recommend developing evidence-based, context-specific public health interventions targeting CL awareness gaps.

**STRENGTHS AND LIMITATIONS OF THIS STUDY**

- This study represents one of the largest surveys assessing community awareness of cutaneous leishmaniasis (CL) in a district with a high incidence rate in Sri Lanka.
- CL disease awareness is operationalised based on a systematic expert consensus approach, thereby distinguishing it from the methodologies used in previous studies.
- The study exemplifies how researchers can use qualitative exploration and community and multisectoral involvement to design and execute a survey effectively.
- The proportion of those who perceived CL as curable could be overestimated because the question was not specified as ‘curable with biomedical treatment’.

## Introduction

 Leishmaniasis, a neglected tropical disease, is caused by *Leishmania* protozoa transmitted to humans by infected female sandflies.^1^ Leishmaniasis shows a high incidence in Asia, Africa, Central and South America and the Mediterranean,^1^ with an estimated 0.7–1.0 million annual incidences worldwide.^2^ Among the three primary clinical forms of leishmaniasis, namely cutaneous (CL), mucocutaneous (MCL) and visceral leishmaniasis (VL),^2^ CL is the most prevalent form, causing long-lasting skin lesion(s) leading to disfigurement and scar formation.^3^ Biomedical treatment for CL is available,^4^ and early treatment minimises the disease’s complications and psychosocial burden.^5^ Early detection and treatment are key disease control methods, given the absence of prophylaxis or vaccination, limited evidence and resources for primary prevention, including vector control.^6^ Public awareness of CL is crucial for a proactive approach to seeking healthcare.^5^

A lack of disease awareness may result in an abundance and proliferation of explanatory assumptions, contributing to behaviours such as self-treatments and delays in biomedical healthcare-seeking.^7 8^ Delays in seeking healthcare for CL^9 10^ result in increased disease severity, complications like lesion dissemination or secondary infections, increased risk of disease transmission and higher burden to the affected people and healthcare systems.^911^,^13^ However, community awareness of CL is reported to be low^8 9 14 15^ and masked by socially constructed myths and beliefs in many countries,^5 7 8^ which may reflect the amount of neglect at the public health policy level. Several problems might be associated with assessing and reporting disease awareness, such as a tendency of research participants to make speculations to appear knowledgeable and well-informed.^16^ Occasionally, the researchers also interpret ‘disease awareness’ as merely having heard of the disease name or asking questions related to pathogen and vector biology, which may not directly trigger people’s healthcare-seeking. The outcome is inaccurate interpretations and conclusions derived from the findings, neglecting the complexity of disease awareness and its broader implications on public health.

Sri Lanka has reported leishmaniasis cases since 1904.^17^ The disease was included in the national surveillance system for communicable diseases as a notifiable disease in 2008 with the re-emergence of the disease.^18^ CL is the most prevalent form of leishmaniasis in Sri Lanka and is unusually caused by the parasite *Leishmania donovani,* which is responsible for VL in other countries such as India.^19^ Several locally acquired VL and MCL cases have been reported sporadically in Sri Lanka,^14^ but skin-related pathology is most common. From 2001 to 2019, more than 19 000 leishmaniasis cases were reported in Sri Lanka. The highest mean annual incidence rate of CL was recorded from the dry zones in the Northern and Southern parts of the country, namely Hambantota, Anuradhapura, Polonnaruwa, Matara and Vavuniya, respectively.^20^ Apart from the availability of healthcare facilities, delayed healthcare-seeking in CL in Sri Lanka has been reported,^21 22^ which might be associated with a lack of disease awareness among the general population. Very few studies have been conducted to assess community awareness of leishmaniasis in Sri Lanka during the past two decades. These studies show contrasting findings with different levels of CL awareness in endemic settings including Anuradhapura (2009–2012, 2016–2017), Matara and Kurunegala (2013–2016) districts^23^,^27^ and non-endemic local settings such as Delft Island (2005),^28^ while a recently published (2022) case–control study shows a high level of knowledge of the name of the vector, symptoms, and curability of CL.^29^ These findings denote the lack of bespoke studies to identify the actual gaps in CL awareness that are particularly tailored to the unique sociocultural context of Sri Lanka. In this paper, we describe the assessment of CL awareness and perceptions among the public in a disease-endemic region in Sri Lanka to address identified challenges in the view of informing public health policy.

## Methods

### Study design

We conducted a population-based, descriptive, cross-sectional household survey to assess CL awareness and perceptions among the endemic community residing in the Anuradhapura district, Sri Lanka. This study is part of a multicountry (Brazil, Ethiopia, Sri Lanka and the United Kingdom) global health research programme entitled, ‘Empowering people with Cutaneous Leishmaniasis: Intervention Programme to improve the patient journey and reduce Stigma via community Education’ (ECLIPSE).

### Study setting

Anuradhapura district is the largest district in Sri Lanka by total land area. It is divided administratively into 22 Divisional Secretariat (DS) divisions and 694 Grama Niladhari (GN) divisions. The DS division is Sri Lanka’s third-level administrative division, while the GN division is its smallest administrative unit. Each DS division in the Anuradhapura district comprises 15–53 GN divisions.^30^ As of 2019, the total population and number of households in the Anuradhapura district were 929 539^31^ and 246 578,^32^ respectively. The majority of the population in the Anuradhapura district belongs to the rural sector (94.6%). The main ethnic group is Sinhalese (91%), followed by Sri Lanka Moor (8.2%), Tamil (0.6%) and others (0.2%).^33^ Among the economically active population within the district, 52.6% are employed, and the majority are own account workers (self-employers) (48.7%), government (20.8%) or private sector employees (15.9%). The mainstay of the economy in the district is agriculture.^34^ Over the past decade, the Anuradhapura district has recorded the second-highest mean annual incidence rates of leishmaniasis (25.46 per 1000 population) among the other districts such as Hambantota (41.82 per 1000 population), Polonnaruwa (21.78 per 1000 population), Matara (19.26 per 1000 population) and Vavuniya (9.16 per 1000 population).^20^ In 2023, 738 leishmaniasis cases were reported in Anuradhapura district out of the 3721 total leishmaniasis cases reported in Sri Lanka.^35^ In the Anuradhapura district, diagnostic services for CL are available only at specific government base hospitals (secondary care) and the teaching hospital (tertiary care). However, treatment for CL is exclusively administered at three designated hospitals within the district: Anuradhapura Teaching Hospital, Thambuttegama Base Hospital and Padaviya Base Hospital.

### Study population and sample size calculation

Our study population was adults (≥18 years) who permanently resided in the Anuradhapura district during the data collection period. Any person unable to comprehend and/or respond to the questions was excluded from the survey. The sample size was calculated using the standard sample size calculation formula for cross-sectional studies with finite populations, assuming the study population size of 700 000,^36^ anticipated awareness level of leishmaniasis of 60%,^27^ absolute precision of 4%, design effect of 2.5 and critical value at 95% confidence level as 1.96. After adding a 10% non-response rate, the total sample size was calculated as 1584.

### Sampling technique

We used a multistage cluster sampling technique with probability proportional to size. We included all the 22 DS divisions in the Anuradhapura district within the sampling framework. A cluster was defined as 10 households per GN division. In the first stage, we calculated the number of GN divisions to be selected from each DS area according to the population size. In the second stage, GN divisions were selected using a simple random sampling technique using the Excel-based random number generation method. A total of 158 GN divisions within the district were selected. In the third stage, we selected 10 households from each GN division. The GN office or community hall (in situations where the GN office was located out of the village) of each GN division served as the focal point for household selection, and 10 consecutive households located on either side of the road were approached. Online supplemental file 1 summarises the household selection process of the survey.

### Patient and public involvement

We involved CL patients and the public in designing the survey questionnaire to ensure its relevance and comprehensiveness. Detailed information regarding their involvement can be found in the ‘Development and pilot testing of the questionnaire’ section. We have disseminated the study findings to patients and the public via local media, community meetings, reports and presentations.

### Development and pilot testing of the questionnaire

The questionnaire development and pilot testing process were supported and refined by community engagement and involvement (CEI). We developed an Interviewer-Administered Questionnaire for data collection according to standard guidelines.^37^ We incorporated several approaches in questionnaire development to ensure scientific validity and cultural appropriateness. We reviewed existing literature to generate questionnaire items. We modified the items and terminology based on the findings of a multimethod ethnographic study conducted within the same study setting (Details of the ethnographic study conducted by the ECLIPSE Sri Lanka researchers have been published elsewhere).^13 21 38^ Eight key informant interviews with purposefully selected participants from the health, agriculture and administration sectors provided feedback on the questionnaire items, design and piloting. Finally, we consulted experts in the fields of public health, parasitology, health promotion, mass communication, sociology and medical anthropology to assess the validity of the content of the questionnaire.^37^

We then conducted a pilot study in 100 households in a separate community with a similar demographic and sociocultural context to the study setting. A group of volunteer community members, including people with the disease, was involved in planning, selecting households and introducing the data collectors to each household. Following this pilot study, we held a review meeting with the data collectors and community members. Concerns raised regarding the understandability of the intended meanings of some questions, the responses and the length of the questionnaire were addressed. We also prepared a detailed interviewer guide to minimise potential interviewer bias.

### Variables and operationalising ‘disease awareness’

The three-part questionnaire included (1) the study participants’ sociodemographic data (gender, age, marital status, ethnicity, religion, education and income-generating activities), (2) the variables for assessing disease awareness and perceptions of CL (disease name(s), clinical manifestations, transmission, whether it can be cured, where to seek healthcare and perceived susceptibility for CL) and (3) data collectors’ observations on participants’ environmental conditions (housing structure and presence of susceptible environmental conditions surrounding the household).

In operationalising variables, we focused on what should constitute ‘CL disease awareness’ sufficient to state that people are aware of the disease. Based on the experience from the previous ethnographic study^21^ and expert opinion, we defined a participant to have CL disease awareness if they were aware of three basic facts of the disease: (1) aware of the correct name (leishmaniasis) or locally used term of the disease (*wæli mæssāgē leḍē*: *wæli* is equivalent to sand, *mæssā* is equivalent to fly and *leḍē* is equivalent to disease)*,* (2) aware that the sandfly transmits the disease and (3) aware that the disease is characterised by a long-lasting skin lesion(s).

### Data collection

Data collection was conducted in August–September 2022. Seven trained research assistants with experience in data collection for public health studies in the same field setting collected the data. All the data collectors were competent in understanding, speaking and writing Sinhala (the main native language). One was competent in using both Sinhala and Tamil (the language used by the Tamil and Moor ethnic groups). Data collectors completed the questionnaire using a face-to-face interview with each participant. In each selected household, they invited the primary healthcare-related decision-maker (who mainly makes health-related decisions in the household) to participate in the study. If that member was absent, the secondary healthcare-related decision-maker in the household was invited. If both members were absent, a household was permanently vacant, or occupants declined to participate, the data collectors moved on to the next household. For the respondents who had heard the correct or locally used name of leishmaniasis, completing each questionnaire took 20–25 minutes, if not 10–15 minutes, as there were fewer questions to ask.

### Data analysis

We manually entered and cleaned the data in Microsoft Excel Spreadsheet and then imported it into SPSS (V.29.0) for analysis. Data included in 20% of the questionnaires were rechecked manually to ensure the quality of the data entry process. Sociodemographic data, environmental conditions, awareness of the disease name, transmission, clinical manifestations, appropriate treatment centres and perceptions towards curability and susceptibility of CL were analysed using descriptive statistics, including counts, percentages, and mean and standard deviation (SD). The Chi-square test was used to test whether there was any statistical significance between different associated factors and awareness-related variables.

## Results

### Sample characteristics

**Figure 1:**
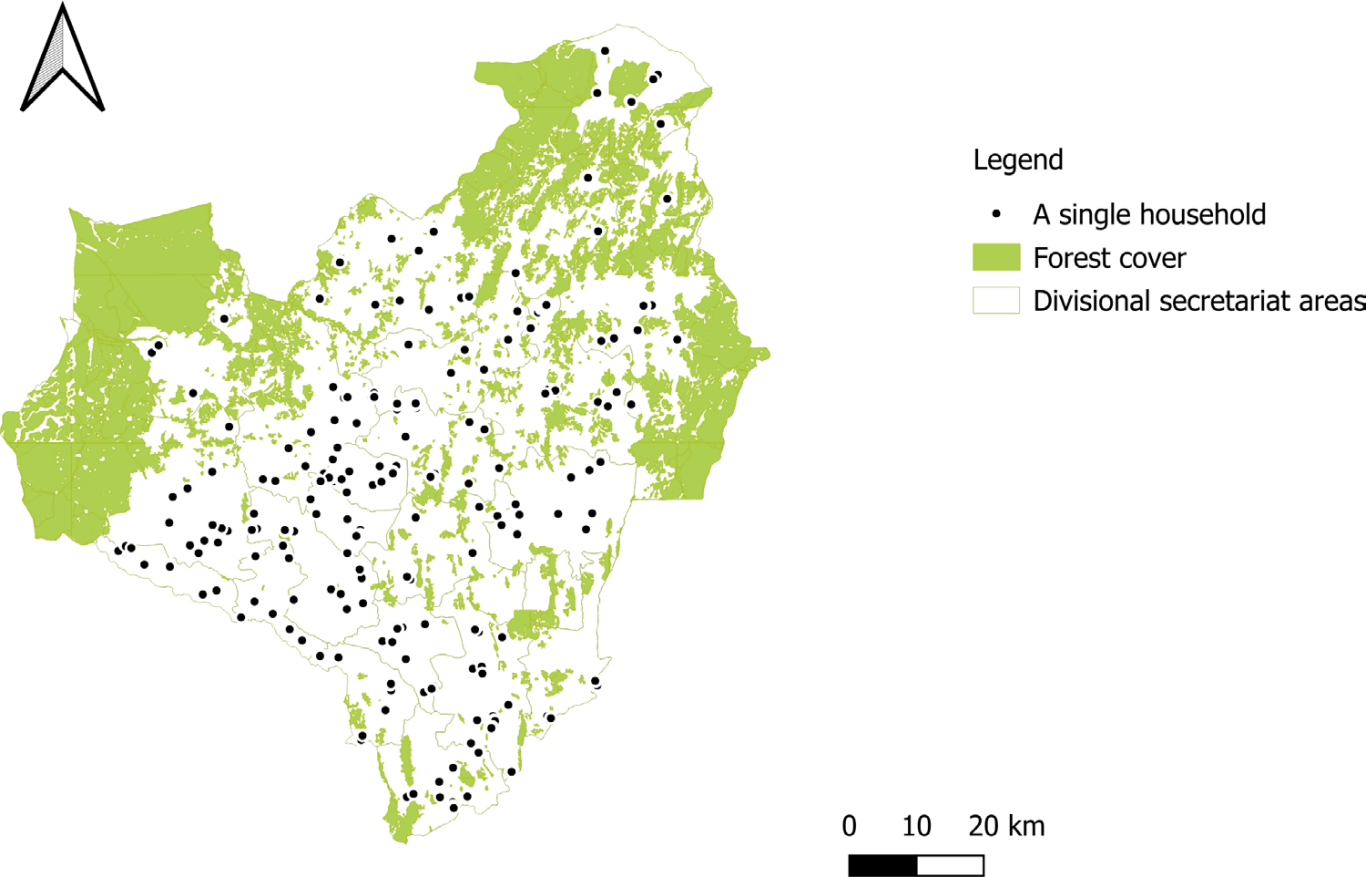
The spatial distribution of households participated in the survey in the Anuradhapura district of Sri Lanka.

Of the 1580 households invited, respondents from 1555 (98.4%) households participated in this study (figure 1). The remaining households (n=25, 1.6%) declined to participate due to the general dislike of participating in surveys or the necessity of leaving the household for other matters. In 1058 (68.0%) households, females were the primary healthcare-related decision-makers. Of those, 977 (92.3%) females were available for the interview. In the remaining households where the primary healthcare-related decision-maker was a male (n=497, 32.0%), 360 (72.4%) were available for the interview. Additionally, 218 (14.0%) respondents served as secondary healthcare-related decision-makers, including 180 (82.6%) females and 38 (17.4%) males. The age of the respondents ranged from 18 to 87 years (mean=48.2 years, SD=13.8). Nearly 95% of the individuals belonged to the Sinhalese ethnic group. The majority of the respondents were the most educated person in the household (n=863, 55.5%). The average household size was 3.9 (SD=1.5). Table 1 shows the detailed sample characteristics. Twenty-four (1.5%) reported that they were treated for leishmaniasis/*wæli mæssāgē leḍē*, while 150 (9.6%) reported that a family member/close friend (close contact) had leishmaniasis, and 98 (6.3%) reported that a person known to them (not a close contact) had leishmaniasis.

**Table 1:** Sample characteristics and CL disease awareness

Characteristic	N (%)	Number with CL disease awareness* (%)
Gender (n=1555)		
Female	1157 (74.4)	44 (3.8)
Male	398 (25.6)	12 (3.0)
Age (years) (n=1555)		
18–24	43 (2.8)	1 (2.3)
25–54	985 (63.3)	45 (4.6)
55–64	311 (20.0)	7 (2.3)
≥65	216 (13.9)	3 (1.4)
Ethnicity (n=1555)		
Sinhalese	1479 (95.1)	56 (3.8)
Moor	71 (4.6)	0 (0.0)
Tamil	5 (0.3)	0 (0.0)
School education of the participant (n=1555)		
No schooling	15 (1.0)	1 (6.7)
Grades 1–5	155 (10.0)	3 (1.9)
Grades 6–11	935 (60.1)	32 (3.4)
Grades 12–13	450 (28.9)	20 (4.4)
Highest education level of the most educated person in the household (n=1550)		
School education only	1144 (73.8)	43 (3.8)
Tertiary education	406 (26.2)	12 (3.0)
The main occupation of the participant (n=1555)		
Farming	301 (19.4)	13 (4.3)
Non-farming	1254 (80.6)	43 (3.4)
Availability of regular monthly income for the household (n=1555)		
Yes	817 (52.5)	27 (3.3)
No	738 (47.5)	29 (3.9)
Housing structure and environmental conditions (n=1555)		
Type of housing (based on roofing, floor and wall materials)		
Permanent	842 (54.1)	25 (3.0)
Semipermanent	635 (40.8)	29 (4.6)
Improvised	78 (5.0)	2 (2.6)
Presence of wet/dark/cold places surrounding the household† (up to 20 m radius)		
Forestry area		
Yes	650 (41.8)	24 (3.7)
No	905 (58.2)	32 (3.5)
Stagnant water bodies		
Yes	96 (6.2)	3 (3.1)
No	1459 (93.8)	53 (3.6)
Paddy fields		
Yes	125 (8.0)	8 (6.4)
No	1430 (92.0)	48 (3.4)
Banana cultivations		
Yes	397 (25.5)	13 (3.3)
No	1158 (74.5)	43 (3.7)
The presence of an animal stall/farm close to the household (up to 20 m radius)		
Yes	202 (13.0)	6 (3.0)
No	1353 (87.0)	50 (3.7)

* CL disease awareness was defined as having heard of the correct name (leishmaniasis) or locally used term of the disease (*wæli mæssāgē leḍē*) and being aware that the sandfly transmits the disease and long-lasting skin lesion(s) as the primary disease characteristic.

† Percentage exceeds 100 due to multiple responses.

CLcutaneous leishmaniasis

### CL disease awareness

According to our operationalised definition, only 3.6% (n=56) of the respondents had CL disease awareness (table 1). We could not observe any significant difference (p>0.05) in CL disease awareness according to the sociodemographic, economic, housing and environmental conditions-related variables (online supplemental file 2).

**Figure 2:**
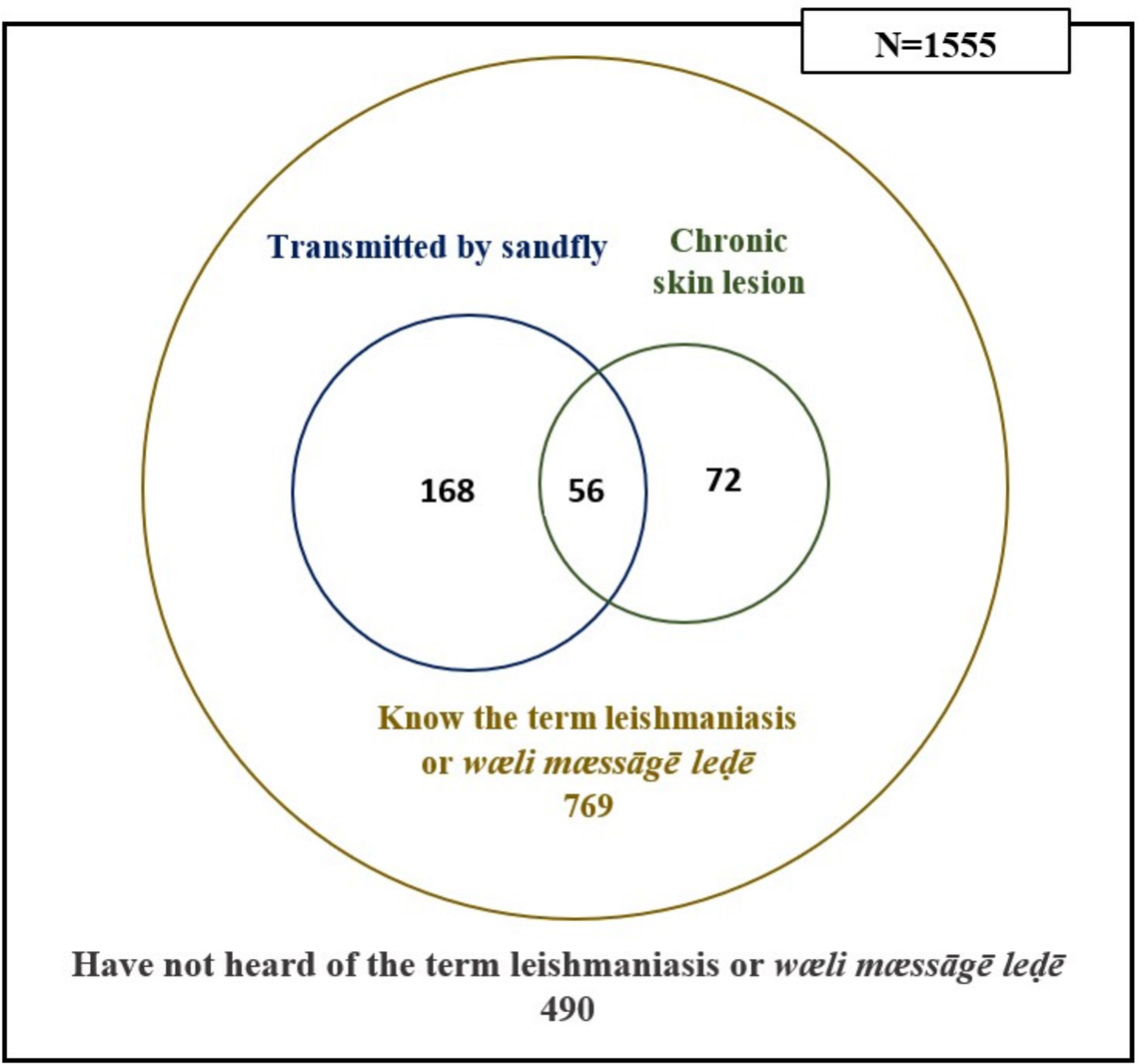
Venn diagram summarising the data relevant to CL disease awareness. CL, cutaneous leishmaniasis.

Out of the total study sample, only 142 (9.1%) respondents had heard of the term ‘leishmaniasis’. Among the respondents who had heard either the correct name (leishmaniasis) or the locally used term for the disease (*wæli mæssāgē leḍē*) (n=1065, 68.5%), the majority did not know the method of disease transmission and its clinical manifestations. Only 224 (21.0%) knew the vector of leishmaniasis, and 128 (12.0%) knew the primary characteristic of CL (long-lasting skin lesion(s)) (figure 2). Of the respondents who had heard the disease name (n=1065), the majority had heard it from a friend or villager (n=263, 24.7%) or could not specify the source (n=249, 23.4%). Others had heard of it from television (n=176, 16.5%), a family member/relative who had leishmaniasis (n=132, 12.4 %), a hospital or medical officer of health office (n=107, 10.0%), another person with leishmaniasis (n=88, 8.3%), leaflets (n=32, 3.0%), YouTube (n=26, 2.4%), newspapers (n=20, 1.9%) and radio (n=8, 0.8%).

**Figure 3:**
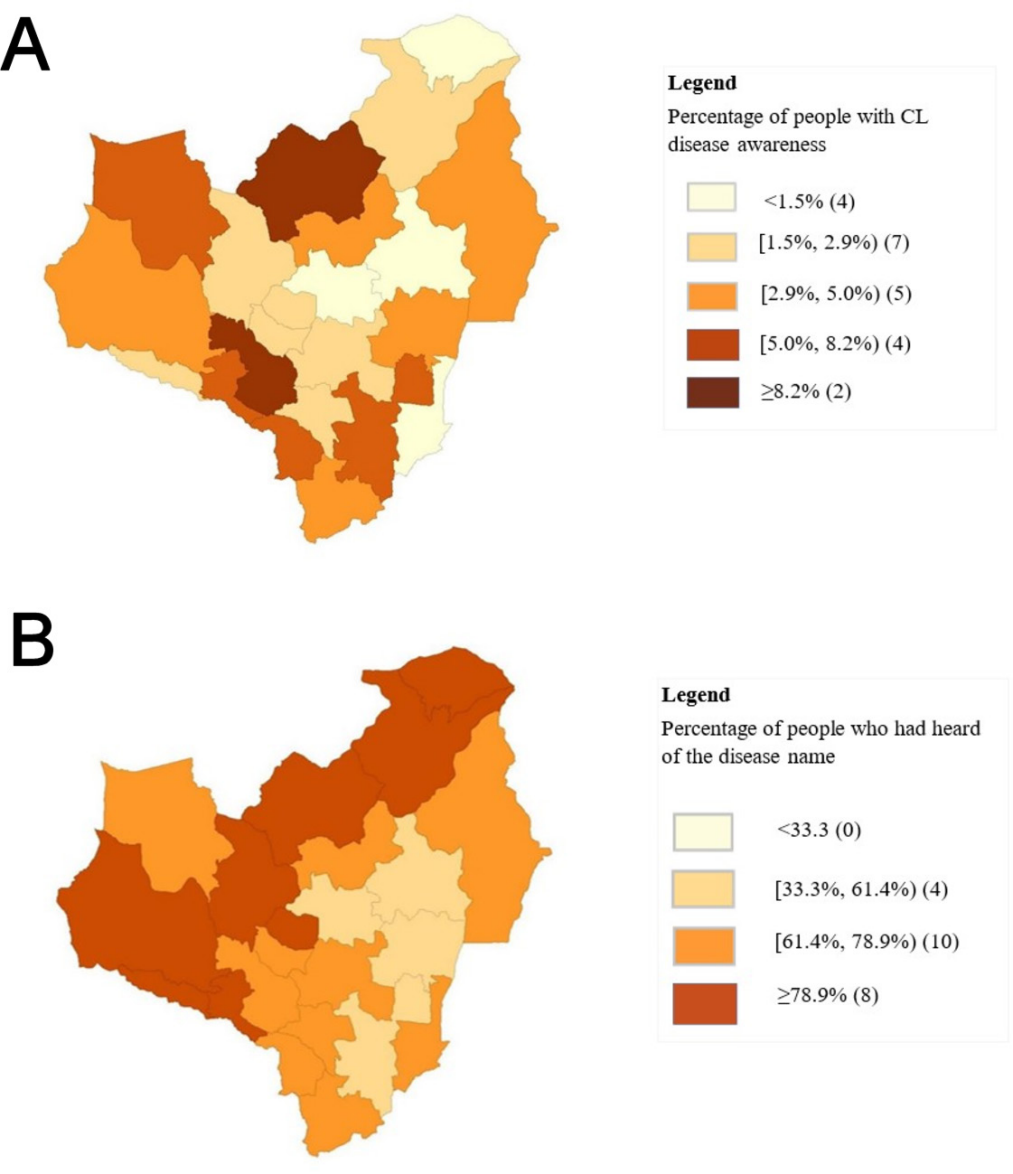
(A) Differences in the CL disease awareness across the 22 DS areas in the Anuradhapura district, Sri Lanka. (B) Differences in the awareness of the disease name across the 22 DS areas in the Anuradhapura district, Sri Lanka. CL, cutaneous leishmaniasis; DS, Divisional secretariat.

Even though the percentage of respondents with CL disease awareness differed across all 22 DS areas in the Anuradhapura district, these differences were not statistically significant (χ^2^=27.593, df=21, p=0.152) (figure 3A). However, the awareness of the correct or local name of the disease significantly differed between the DS areas (χ^2^=184.635, df=21, p<0.05). In contrast to the CL disease awareness, more than 50% of the respondents residing in most of the DS areas in the Anuradhapura district had heard at least the name of the disease (figure 3B).

### Other aspects of awareness and perceptions of CL

Of the respondents who had heard the disease name (n=1065), 453 (42.5%) perceived that CL is curable with treatment, while 522 (49.0%) were aware of such treatment centres and 428 (41.0%) respondents, excluding people with CL perceived that they could be susceptible for CL. Among the respondents with CL disease awareness (n=56), 42 (75.0%), 54 (96.4%) and 44 (88.0%) perceived that CL is curable, were aware of treatment centres and perceived that they could be susceptible to CL, respectively.

### Differences in the awareness and perceptions related to CL between the subgroups

**Figure 4:**
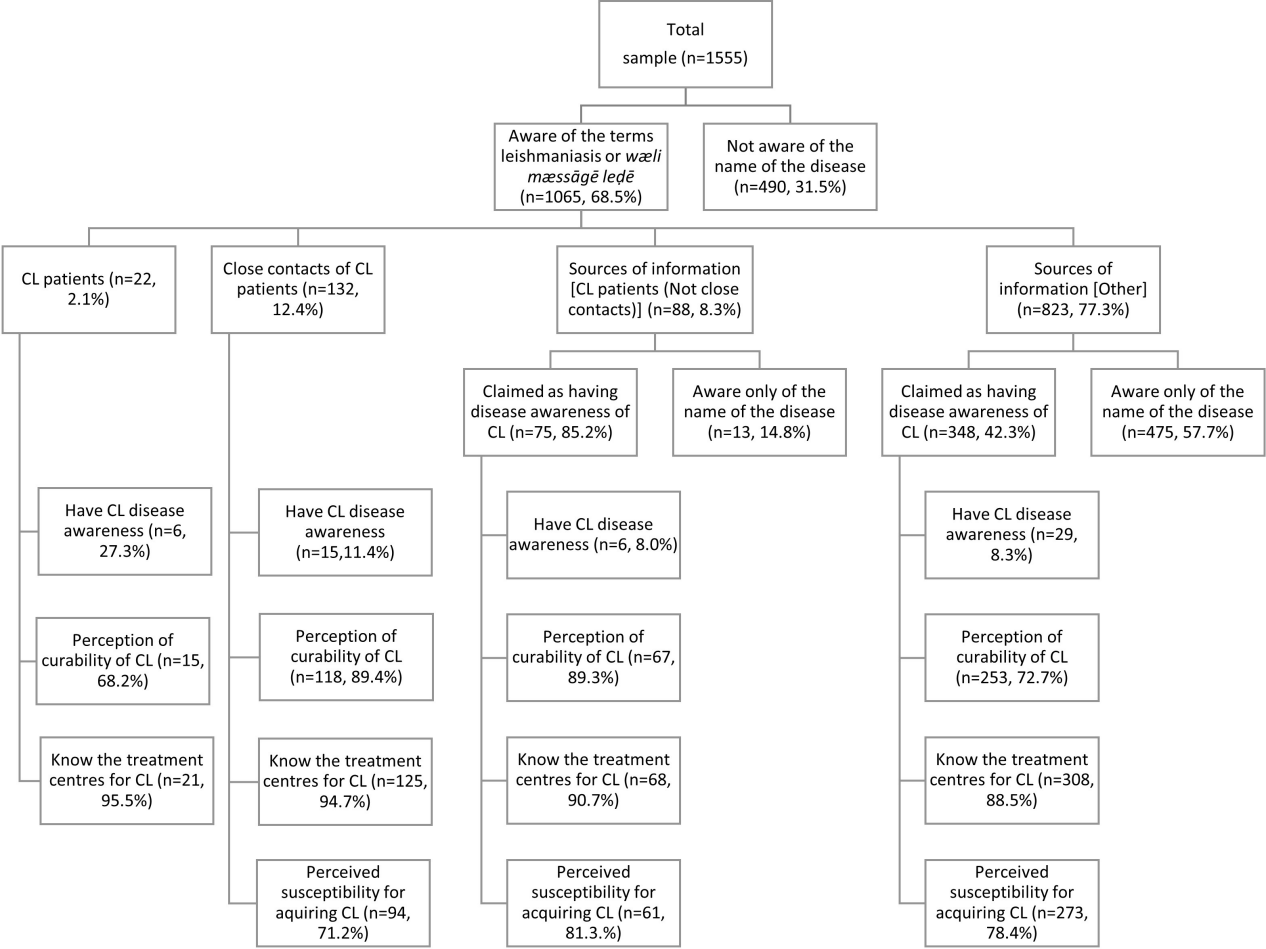
Differences in the awareness and perceptions related to CL between the subgroups of the study sample. Awareness of the vector, primary disease characteristics, curability, treatment centres and perceived susceptibility for CL was not assessed for the respondents who did not know about the disease other than its name. CL, cutaneous leishmaniasis.

Figure 4 illustrates the difference in the aspects of awareness and perceptions of CL among the subgroups out of those who had heard of the disease: (1) people with active CL or had previously had the disease, (2) people who had a family/close friend with CL, (3) people who knew a person with CL (not a close contact) and (4) people who came to know about the disease from other sources. There was a significant difference in the percentage of respondents with CL disease awareness among these subgroups (χ^2^=40.785, df=3, p<0.05), indicating that people with CL and close contacts of those with CL were more aware of the disease than the other two subgroups.

### Self-reported disease awareness of CL

We assessed the self-reported disease awareness of CL only from the people who had not had CL nor were close contacts of people who had CL (n=911). For that, we asked whether they had heard only of the disease name or anything new more than the disease name (‘knew something beyond the disease name’ or ‘knew very well about the disease’). Of those who claimed that they ‘knew something beyond the name of the disease’ (n=364, 40.0%), only 28 (7.7%) had CL disease awareness as defined above, whereas among those who claimed that they were ‘well aware’ (n=59, 6.5%), only seven (11.9%) had CL disease awareness (χ2=44.384, df=2, p<0.05).

### Local terminology and disease awareness

Within the total study sample, there were 196 (12.6%) respondents who had heard another local term *wæli mækkāgē leḍē*. This term is ambiguously used in the local context, where some people refer to a disease caused by a flea living in the sand while others refer to leishmaniasis.^26^ Two people with CL (1.0%), 18 close contacts of people with CL (9.2%), and 176 respondents who were neither people with CL nor close contact with people with CL (89.8%) had only heard the term *wæli mækkāgē leḍē* and not leishmaniasis or *wæli mæssāgē leḍē*. Among them*,* only one respondent (0.5%) accurately identified both the vector and the long-lasting characteristic of the skin lesion in CL. Therefore, those who had heard of the term *wæli mækkāgē leḍē* in this study may not have interpreted it as leishmaniasis; instead, they have interpreted it as another disease.

## Discussion

This study represents one of the largest surveys to assess community awareness of CL in Sri Lanka. Our study findings clearly show that a significantly lower proportion of the population (3.6%) had CL disease awareness. Nearly three-quarters of the people with CL were not aware of the disease. Despite the self-reported awareness beyond the name of the disease, fewer respondents possessed actual CL disease awareness. The findings indicate that attempts to increase public and patient awareness of CL have not been successful so far in the district.

Disease awareness is imperative for controlling re-emerging infectious diseases by enabling early case detection, rapid response and effective prevention measures. Many studies, including leishmaniasis-related research, often measure disease awareness without providing explicit definitions.^15 39 40^ A cross-sectional survey conducted in Matara district, Sri Lanka, involving 120 inhabitants has concluded a ‘lower level of CL awareness’ with 21.7% of participants having heard of the term ‘leishmaniasis’ and 68.1% having heard of the sandfly as the vector.^25^ Claiming a ‘low level of CL awareness’ despite the relatively high figures reported is questionable within their study. A recent study conducted in a highly disease-prevalent area in the Anuradhapura district reported that 75.1% ‘had knowledge of CL’,^24^ while another study conducted in the Matara district, Sri Lanka declared that 18.4% had ‘CL disease awareness’^41^ without specifying which aspects comprised the CL knowledge or awareness. Furthermore, a nationwide case–control study conducted in Sri Lanka reported a ‘satisfactory level of basic knowledge of CL’ among the majority of the participants based on a predetermined scoring system.^29^ However, it is noteworthy that only 1.4% of the participants recognised the term ‘leishmaniasis’, and many identified CL merely as a skin lesion without specifying its distinct characteristics. Interestingly, the proportion of respondents with CL disease awareness in our study increased from 3.6% up to 8.4% when the awareness of the long-lasting characteristic of CL is excluded and the mere awareness of CL as a skin lesion (papule/nodule/wound) is included. From the public health perspective, the ‘long-lasting’ skin lesion will lead a patient to seek healthcare. Yet, most of the local studies, including the aforementioned nationwide survey, have investigated the existence of a mere ‘wound’,^24^ ‘acne’^25^ or ‘skin lesion’^29^ as the symptoms of CL, while some studies just reported ‘disease symptoms’ were ‘known’ or ‘unknown’^23^ without specifying which types of symptoms they considered in those studies. These findings and methodologies imply that the researchers must be vigilant in defining CL disease awareness and its components and accurately interpreting and comparing the findings across different studies.

Significant differences in CL knowledge among different gender and age categories have been reported in studies conducted in Ethiopia^42^ and Yemen.^43^ Even though we could not observe such differences in our study, the disease awareness of people with CL and their close contacts was significantly higher than non-close contacts, which was both in line^29^ and in contrast with other local study findings.^44^ Nonetheless, an unpromisingly lower level of disease awareness among people with CL in our study (n=6, 27.3%) reflects the extent of the problem, which could be due to the absence or ineffective health education attempts taking place in the local healthcare settings. Therefore, a clear and straightforward health message to inform the patients and public about the existence of such a disease, its transmission, and its primary clinical characteristic and then advising them to seek biomedical treatment would suffice in the initial phase of a well-designed public awareness campaign. Although a centrally mediated public health system is operating within the district, we could observe significant disparities in having heard of the disease name across the DS areas in Anuradhapura, which could be associated with the disease incidence, availability of CL treatment centres (ie, Padaviya, Nuwaragam Palatha East, Thambuttegama DS areas in Anuradhapura) or public health interventions carried out in those areas.

Interestingly, most of the respondents with CL disease awareness perceived that CL is curable (n=42,750.0%), were aware of treatment centres (n=54, 96.4%), and perceived that they could be susceptible to CL (n=44, 88.0%). Yet, of the general population who had heard the name of the disease, half were not aware of curability or treatment centres and perceived that they could get the disease. This implies that people with CL disease awareness knew more specific aspects of awareness, which could trigger healthcare-seeking for CL. CL case detection in Sri Lanka primarily occurs through patients voluntarily reporting themselves to healthcare facilities, with only a limited number of screening programmes being conducted.^45^ CL is treated only in government hospitals where dermatologists are available, limiting the healthcare institutions to one or two per district, particularly in rural areas with susceptible populations.^45^ While only one person with CL in our study sample had recommended Ayurvedic or traditional treatment for CL, other studies conducted in Ethiopia^5 46^ and Iran^47^ indicate that many participants believe that traditional medicine would be the best option for treating CL and thus not seeking biomedical healthcare. This finding may be due to the contextual differences in accessibility, affordability and acceptability of different healthcare systems in different countries.

Responses to health-related inquiries from individuals can be influenced by factors such as social desirability bias and cultural orientation. As a result, people might lean towards giving positive answers, particularly regarding public health issues, such as asserting familiarity with diseases, even if their knowledge is limited.^48^ In line with that, most of our respondents were confident about their knowledge of the disease, which was often incorrect or misinformed. This could lead to negative impacts in the future, such as delayed healthcare-seeking, continued disease transmission, and ineffective public health interventions. However, the persistent lack of CL awareness, which was identified a decade back from a study conducted in the same study setting,^27^ suggests a lack of effectiveness of awareness programmes or the strategies employed. However, the scenario differs for various diseases in Sri Lanka, as exemplified by the successful control of leprosy in the past through effective and evidence-based social marketing campaigns.^49^ Hence, we must revisit and reevaluate the existing strategies to improve awareness of leishmaniasis in Sri Lanka.

This study has several strengths and limitations. A geographically representative sample coupled with the multistage cluster sampling might increase the study’s external validity, leading to increased generalisability of the findings compared with previous studies conducted in the Anuradhapura district and Sri Lanka. The selection of key health-related decision-makers in the households further increases the public health importance of this study. The hierarchical structure of decision-making in families plays a crucial role in healthcare-seeking behaviour.^50^ Consequently, the knowledge of these decision-makers regarding diseases directly influences the healthcare choices of other family members. This underscores the importance of focusing on the primary healthcare decision-maker(s) within the family for our research. We used ethnographic findings and the CEI approach for survey questionnaire development. These were crucial in understanding the local terminology related to the disease, developing context-specific options for closed-ended questions (ie, options for disease causation, symptoms and curability) and determining the best period for conducting the survey. Operationalisation of CL disease awareness also enabled accurate interpretation and cross-cultural comparison, which led us to reveal the precise picture of leishmaniasis awareness in an endemic region of the country. Furthermore, we provided the data collectors with a detailed interviewer guide to reduce interviewer bias when delivering the questions. There could be a possible overestimation of the proportion of those who perceived CL as curable, given that the question was not specified as ‘curable with biomedical treatment’.

Our study showed that awareness of CL is very low in the disease-endemic Anuradhapura district in Sri Lanka. The credibility of mere awareness of having heard of leishmaniasis and people’s perception that they know about CL is questionable within this population. Therefore, we recommend developing context-specific and evidence-based public health interventions to improve community awareness of CL, revisiting the effectiveness of existing public awareness campaigns and strengthening the role of healthcare institutions in enhancing awareness of CL patients using effective strategies.

## supplementary material

10.1136/bmjopen-2024-088714 online supplemental file 1

10.1136/bmjopen-2024-088714 online supplemental file 2

## Data Availability

Data are available on reasonable request. All data relevant to the study are included in the article or uploaded as online supplemental information.
