# Supplementary figures and images for: Investigating disease awareness of cutaneous leishmaniasis in rural Sri Lanka to inform public health services: a cross-sectional study
Source: BMJ Open. 2024 Nov 24;14(11):e088714. doi: 10.1136/bmjopen-2024-088714 (PMC11590865; doi:10.1136/bmjopen-2024-088714)

## Supplementary File 1

Summary of the household selection process of the survey

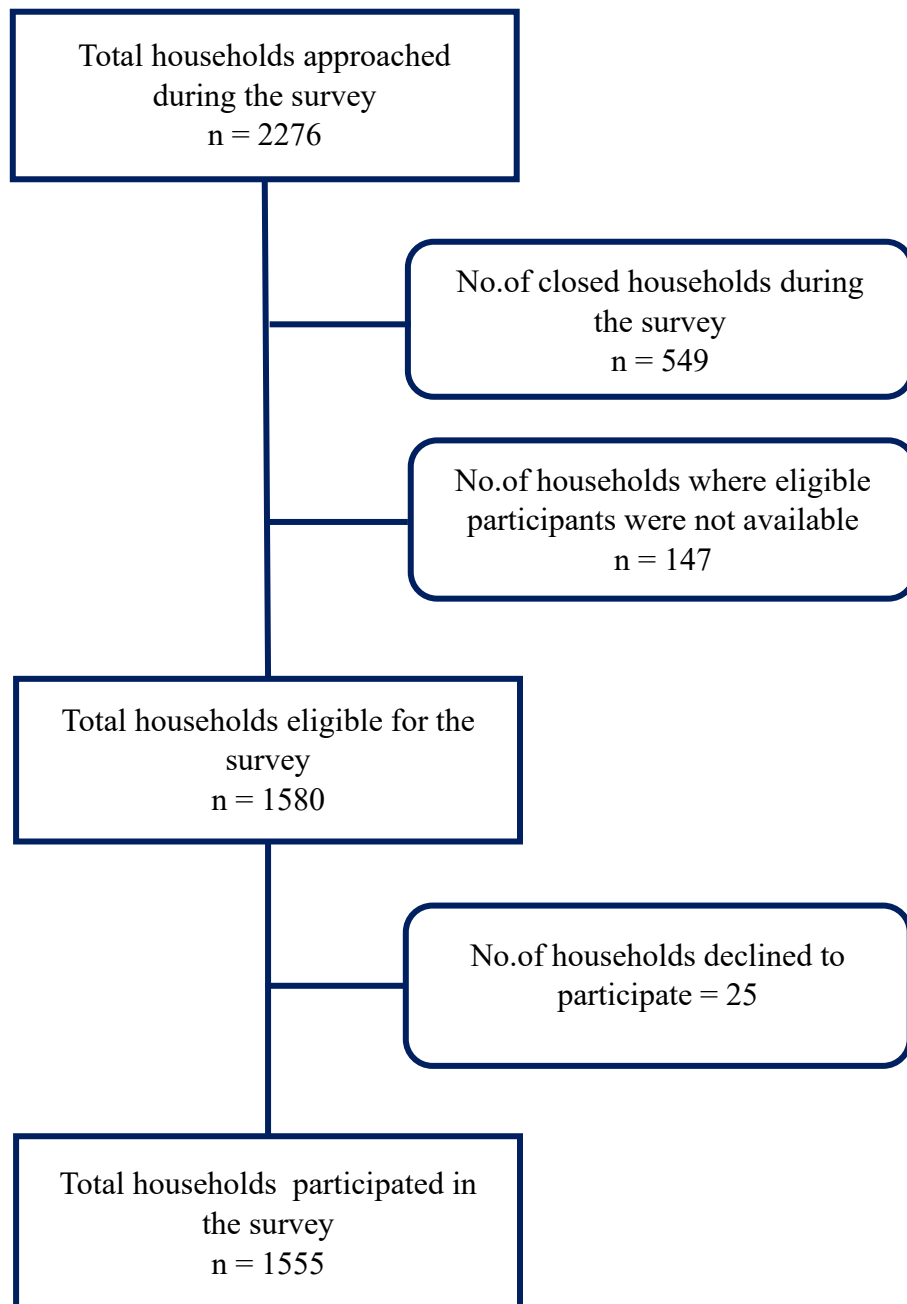

Supplement: online supplemental file 1 [file bmjopen-14-11-s001.pdf]
