# Supplementary material for: Investigating disease awareness of cutaneous leishmaniasis in rural Sri Lanka to inform public health services: a cross-sectional study
Source: BMJ Open. 2024 Nov 24;14(11):e088714. doi: 10.1136/bmjopen-2024-088714 (PMC11590865; doi:10.1136/bmjopen-2024-088714)
Supplement: online supplemental file 2 [file bmjopen-14-11-s002.pdf]

Supplementary File 2

The difference in CL disease awareness based on socio-demographic variables

**Characteristic (N=56)**	**Number with CL disease awareness (%)**	**Chi-square statistics (df), p-value**
Sex		
Female	44 (3.8)	$\chi2$ (1) = 0.529,
Male	12 (3.0)	p = 0.467
Age (Years)		
18-24	1 (2.3)	$\chi2$ (3) = 7.535,
25-54	45 (4.6)	p = 0.057
55-64	7 (2.3)	
≥65	3 (1.4)	
Ethnicity		
Sinhalese	56 (3.8)	$\chi2$ (2) = 2.985,
Moor	0 (0.0)	p = 0.225
Tamil	0 (0.0)	
School education of the participant		
No schooling	1 (6.7)	$\chi2$ (3) = 2.653,
Grade 1-5	3 (1.9)	p = 0.448
Grade 6-11	32 (3.4)	
Grade 12-13	20 (4.4)	
Highest education level of the most educated person in the household		
School education only	43 (3.8)	$\chi2$ (1) = 0.565,
Tertiary education	12 (3.0)	p = 0.452
The main occupation of the participant		
Farming	13 (4.3)	$\chi2$ (1) = 0.554,
Non-farming	43 (3.4)	p = 0.457
Availability of regular monthly income for the household		
Yes	27 (3.3)	$\chi2$ (1) = 0.436,
No	29 (3.9)	p = 0.509
Housing structure and environmental conditions		
Type of housing (based on roofing, floor and wall materials)		
Permanent	25 (3.0)	$\chi2$ (2) = 2.917,
Semi-permanent	29 (4.6)	p = 0.233
Improvised	2 (2.6)	
Presence of wet/dark/cold places surrounding the household* (up to 20 m radius)		
Forestry area		
Yes	24 (3.7)	$\chi2$ (1) = 0.027,
No	32 (3.5)	p = 0.870
Stagnant water bodies		
Yes	3 (3.1)	$\chi2$ (1) = 0.067,
No	53 (3.6)	p = 0.796
Paddy fields		
Yes	8 (6.4)	$\chi2$ (1) = 3.067,
No	48 (3.4)	p = 0.080
Banana cultivations		
Yes	13 (3.3)	$\chi2$ (1) = 0.164,
No	43 (3.7)	p = 0.686
The presence of an animal stall/farm close to the household (up to 20 m radius)		
Yes	6 (3.0)	$\chi2$ (1) = 0.266,
No	50 (3.7)	p = 0.606
