# Supplementary material for: Investigating disease awareness of cutaneous leishmaniasis in rural Sri Lanka to inform public health services: a cross-sectional study
Source: BMJ Open. 2024 Nov 24;14(11):e088714. doi: 10.1136/bmjopen-2024-088714 (PMC11590865; doi:10.1136/bmjopen-2024-088714)
Supplement: Supplementary file 3 [file bmjopen-2024-088714.reviewer_comments.pdf]

PEER REVIEW HISTORY

BMJ Open publishes all reviews undertaken for accepted manuscripts. Reviewers are asked to complete a checklist review form and are provided with free text boxes to elaborate on their assessment. These free text comments are reproduced below.

ARTICLE DETAILS

Title (Provisional)

Investigating disease awareness of cutaneous leishmaniasis in rural Sri Lanka to inform public health services: A cross-sectional study

Authors

Gunasekara, Sonali Dinushika; Agampodi, Thilini; Weerasinghe, Manjula; Fernando, Manoj Sanjeewa; Price, Helen; Wickramasinghe, Nuwan Darshana; Agampodi, Suneth

---

VERSION 1 - REVIEW

**Reviewer**	**1**
**Name**	**Adamu, Andrew**
**Affiliation**	**James Cook University**
**Date**	**17-Jul-2024**
**COI**	**None**

1.Please authors should recast the statement on significance and please if a variable is not significant, the p value should be > 0.05.

2. The study was conducted in Anuradhapura, with a population of about 50,000 people. Can you justify why you use 700,000 people as your population in sample size determination?

**Reviewer**	**2**
**Name**	**Komakech, Allan**
**Affiliation**	**Makerere University**
**Date**	**27-Jul-2024**
**COI**	**None**

---

1. **Title:** include the period the study was done, include study population
2. **Abstract:**

Introduction:

-Knowledge gap is part of awareness, improve the sentence

-Include a burden of leishmaniasis in Sri-Lanka

Methods

-Include the sample size of the study

-Focus on awareness measurement (already there in the text) and not how the tool was developed (as this is not your objective of the study), rather tell us the variables in the tool

Results

-

Conclusion

-Results on awareness and other variables under this are okay

-Page 3, line 50: Result of methodologies assessment do not appear in the results and therefore cannot be used in the conclusion section. Also, this is not your primary objective of the study, please stick to awareness and its sub variables

-Please provide a recommendation towards the low CL

3. **Introduction**
   - Page 5, In line 38 and over the next few lines, you described the methods about data collection and how robust they should be. I would suggest you mention this in your study strengths of the study and defend them there.
   - Page 6, line 24: When were the other studies done? In which years?
   - Page 6, line 29: If you want to leave it information in the introduction about the tools used to study awareness, then you need to show us how different they are from what you are doing. However, I would still suggest pushing this to your discussion
   - Page 6, Line 37: The objective of your study is not to develop a tool but rather to study awareness. Please stick to this. You can then tell us about the robustness of your tool under the discussion (strengths and weaknesses section)
4. **Methods:**
   - Methods are well written;
   - Under study setting, please add any available health services or interventions in this setting
   - Page 7, line 27: in addition to calculating number of cases, calculate incidence per population and evaluate it with other provinces; helps to evaluate burden as well
   - It is important to note that development and testing of a tool is standard procedure
   - Page 10, line 3: how were the 20% questionnaires for rechecking determined?
5. **Results**
   -
6. **Discussion**
   - Page 14, line 26: In this paragraph, you discuss your dependent variable, which is disease awareness. I would suggest that you discuss how the awareness levels were differently assessed for the studies in Sri Lanka. This will show the strength or difference and may explain the disparities

- Page 17, line 17: this is the opportune moment to discuss the advantages of your tool over the others.
- Please describe further how the strengths such as the selection of key health decision-makers is a strengths.
- Page 17, line 44: I think your most important results centre around the low awareness and therefore you should provide recommendations towards messaging to increase awareness as well as a few descriptive variables from the study. Other recommendations such as exploration of the disease and community context before conducting surveys, operationalisation of disease awareness according to standard and rigorous methodologies, the adaptation of the disease awareness assessment questionnaires, using the CEI
- approach and accurate interpretation of the findings in future studies to obtain valid estimates for actual disease awareness were not proven by your study so I would suggest taking them out completely. Focus your recommendations on your conclusions and these conclusions are provided by the results.

7. References

8. Figures

9.

VERSION 1 - AUTHOR RESPONSE

Comments of Reviewer 1

Reviewer comments	Response of authors
Please authors should recast the statement on significance and please if a variable is not significant, the p value should be p> 0.05.	Thank you for the comment. We have reviewed and corrected the significance level throughout.
The study was conducted in Anuradhapura, with a population of about 50,000 people. Can you justify why you use 700,000 people as your population in sample size determination?	The total population in Anuradhapura district is 929,539 (2019), and the population ≥18 years old exceeds 600,000. A reference to justify our study population has been added under the 'study population and sample size calculation section.'

Comments of Reviewer 2

Reviewer comments	Response of authors
Title: include the period the study was done, include the study population	Thank you for the suggestion. The study population and period have been clearly stated in the methods section. Therefore, the authors agreed that it is not necessary to add the study population and study period to the title of the paper.
Abstract: Introduction:<br>Knowledge gap is part of awareness; improve the sentence	Thank you for the comment. As the abstract was modified according to the updated guidelines of the journal, we have removed

	the introduction section from the abstract, which previously included this sentence.
Abstract: Introduction<br>-Include a burden of leishmaniasis in Sri-Lanka	Thank you for the suggestion. As the abstract was modified according to the updated guidelines of the journal, we removed the introduction section from the abstract. However, we have described the burden of leishmaniasis in Sri Lanka in the Introduction section of the paper.
Abstract: Methods<br>Include the sample size of the study	The sample size of the study was included under the results section of the abstract in the original file, and with the updated journal guidelines, we have now included it within the ‘sampling technique and participants’ section of the abstract.
Abstract:Methods<br>Focus on awareness measurement (already there in the text) and not how the tool was developed (as this is not your objective of the study) rather, tell us the variables in the tool	Thank you for the comment. We have removed the sentence on tool development from the abstract, as it is detailed in the methods section of the manuscript.
Abstract: Conclusion<br>Results on awareness and other variables under this are okay	Thank you.
Abstract: Conclusion<br>Page 3, line 50: Results of methodologies assessment do not appear in the results and therefore cannot be used in the conclusion section. Also, this is not your primary objective of the study, and please stick to awareness and its sub-variables	Thank you for the comment. We have removed the conclusions related to the tool development from the abstract.
Abstract: Conclusion<br>Please provide a recommendation towards the low CL	Thank you for the comment. We have included a recommendation on low awareness of CL in the abstract.
Introduction<br>Page 5, In line 38 and over the next few lines, you described the methods about data collection and how robust they should be. I would suggest you mention this in your study strengths of the study and defend them there.	Thank you for the comment. The section that describes the robustness of survey instrument development has been removed from the Introduction section and incorporated into the Discussion section of the manuscript.
Introduction<br>Page 6, line 24: When were the other studies done? In which years?	We specified the study period of previously conducted studies when available.
Introduction	We have removed the descriptions of tools used to study awareness from the

Page 6, line 29: If you want to leave it information in the introduction about the tools used to study awareness, then you need to show us how different they are from what you are doing. However, I would still suggest pushing this to your discussion.	introduction section and incorporated those into the discussion section of the manuscript.
Page 6, Line 37: The objective of your study is not to develop a tool but rather to study awareness. Please stick to this. You can then tell us about the robustness of your tool under the discussion (strengths and weaknesses section)	Thank you for the comment. We have modified the objective of our study accordingly.
Methods<br>Methods are well written.	Thank you.
Methods<br>Under study setting, please add any available health services or interventions in this setting	We have included information on CL diagnostic and treatment services available within the study setting.
Methods<br>Page 7, line 27: in addition to calculating number of cases, calculate incidence per population and evaluate it with other provinces; helps to evaluate burden as well	We have added the incidence rates of leishmaniasis for the districts with the highest mean annual incidence rates in Sri Lanka.
Methods<br>It is important to note that development and testing of a tool is standard procedure	We acknowledge that developing and testing a data collection tool is a standard procedure, and we have combined standard guidelines (reference can be found in the main text under development and pilot testing of the questionnaire) with context-specific techniques to develop a culturally bespoke study instrument to achieve our primary objective.
Methods<br>Page 10, line 3: how were the 20% questionnaires for rechecking determined?	This was determined based on the available time and resources to ensure accuracy in data entry.
Discussion<br>Page 14, line 26: In this paragraph, you discuss your dependent variable, which is disease awareness. I would suggest that you discuss how the awareness levels were differently assessed for the studies in Sri Lanka. This will show the strength or difference and may explain the disparities	This paragraph has been modified by adding and discussing the studies conducted in Sri Lanka and the respective methodologies used to assess CL awareness.
Discussion<br>Page 17, line 17: this is the opportune moment to discuss the advantages of your tool over the others.	We have incorporated strengths in our study instrument in this paragraph on ‘strengths and limitations of the study’ in the discussion section of the manuscript.

Discussion<br>Please describe further how the strengths such as the selection of key health decision-makers is a strengths.	We have described this further in the discussion section.
Discussion<br>Page 17, line 44: I think your most important results centre around the low awareness and therefore you should provide recommendations towards messaging to increase awareness as well as a few descriptive variables from the study. Other recommendations such as exploration of the disease and community context before conducting surveys, operationalisation of disease awareness according to standard and rigorous methodologies, the adaptation of the disease awareness assessment questionnaires, using the CEI approach and accurate interpretation of the findings in future studies to obtain valid estimates for actual disease awareness were not proven by your study so I would suggest taking them out completely. Focus your recommendations on your conclusions and these conclusions are provided by the results.	Thank you for the comment. We have modified the final paragraph on recommendations as per the given comments.

VERSION 2 - REVIEW

Reviewer 2

Name Komakech, Allan

Affiliation Makerere University

Date 16-Oct-2024

COI

The conce
